# Supplementary material for: Being a Parent of Children with Disabilities during the COVID-19 Pandemic: Multi-Method Study of Health, Social Life, and Occupational Situation
Source: Int J Environ Res Public Health. 2023 Feb 10;20(4):3110. doi: 10.3390/ijerph20043110 (PMC9961490; doi:10.3390/ijerph20043110)
Supplement: Supplementary file 1 [file ijerph-20-03110-s001.zip › ijerph-2040800-supplementary.pdf]

Supplemental material

Individual Interview Questions

The following questions relate to the experience you have experienced as parents with one or more children with disabilities in the context of confinement due to the pandemic Covid-19 and you now with the restrictive measures taken in place:

a) Could you fist remind me the categories of deficiencies or disabilities of your child:
01 Vision
02 Hearing
03 Mobility
04 Flexibility
05 Dexterity
06 Pain
07 Learning
08 Developmental / disorder autism spectrum
09 Intellectual
10 Mental health
11 Memory / cognitive
12 Other (please specify) :
99 I prefer not to answer

b) Which diagnosis (s) applies to your child:
01 Traumatic brain injury
02 Spinal cord injury
03 Stroke
04 Amputation
05 Multiple sclerosis
06 Other neurodegenerative disease
07 Autism Spectrum Disorder (ASD)
08 Global development delay
09 Intellectual disability
10 Developmental coordination disorder (DCD)
11 Developmental language disorder
12 Attention deficit disorder with or without hyperactivity (ADHD)
13 Other (please specify):
99 I'd rather not answer

1. Tell me about a typical day for your family during the confinement period, as well as during the current period, where restrictive measures (physical distancing, restriction of movement, closing of daycare centers and schools, suspension of activities of community groups, etc.) have been regulating the pace of your activities for some time?
   - Tell me about your difficulties and the positive elements?
   - Tell me about your family in these troubled times?

2. Tell me about what you said to your child with a developmental disorder (e.g.: intellectual disability, autism spectrum disorder, cerebral palsy, language disorder, hearing loss regarding the pandemic)?
   - How have you explained to your child what the virus is and that it is contagious?
   - What strategy have you developed, for example, to explain to him that he could no longer see his friends?
   - What actions have you taken to facilitate its understanding?

Supplemental material

3. With this inability to carry out regular activities, have you developed a new routine for your child? What is it about?

4. Have you ever felt alone or isolated during the implementation of lockdown in Quebec and the accompanying restrictions? What was your thinking on this subject?
    - Have you called on your entourage, other parents, workers from the CIUSSS de la Capitale-Nationale for respite or a support group of parents such as AIS, Autisme Québec, AQEPA, Dysphasie-Québec, etc.?

5. We know that summer is generally an important season in terms of leisure activities for children in general, and perhaps more particularly for those with disabilities. How was your summer 2020?
    - Was your child able to attend summer camp?
    - What it has involved for you and your family?

6. We sometimes observe that in unusual contexts people are somehow forced to innovate. How has this manifested for you and your family?
    - Have you discovered any resources you didn't think you had?
    - How do you manage to do activities that contribute to the development of your resilience and that generate well-being?

7. How do you see the future for you, your child and your loved ones in a future more or less near, say when social distancing measures will be lifted or even after the end of the Covid-19?

8. Finally, do you have any other concerns or information that you would like to share with us in connection with the Covid-19 pandemic?

Supplemental material

Table S1. Characteristics of the five parents of children with disabilities from Quebec province (Canada) who participated in interview during the COVID-19 pandemic.

**Participant**	**1**	**2**	**3**	**4**	**5**
**Sex**	Woman	Woman	Woman	Woman	Woman
**Age (range), years**	40-49	30-39	50-59	30-39	30-39
**Number of children**	2	2	2	3	2
**Age of children (range), years**	6-11, 12-17	6-11, 6-11	12-17, 12-17	6-11, 6-11, 12-17	6-11, 6-11
**Living as a single parent**	No	No	No	Yes	No
**Attending school, college, CEGEP or university**	No	No	Yes	No	No
**Employment situation**	Full-time salaried employment	Full-time salaried employment	Doing unpaid work (e.g., childcare, volunteering)	Maternity, paternity or parental leave	Full-time salaried employment
**Working in the health and social sector**	Yes	Yes	Yes	Yes	No
